# Impact of washing parameters on bacterial filtration efficiency and breathability of community and medical facemasks

**DOI:** 10.1038/s41598-022-20354-w

**Published:** 2022-09-23

**Authors:** Henrietta Essie Whyte, Aurélie Joubert, Lara Leclerc, Gwendoline Sarry, Paul Verhoeven, Laurence Le Coq, Jérémie Pourchez

**Affiliations:** 1 Mines Saint-Etienne, INSERM, U 1059 Sainbiose, Centre CIS, Université Lyon, Université Jean Monnet, 42023 Saint-Étienne, France; 2 IMT Atlantique, CNRS, GEPEA, UMR 6144, 4 rue Alfred Kastler, 44307 Nantes, France; 3 CIRI (Centre International de Recherche en Infectiologie), GIMAP Team, INSERM, U1111, CNRS UMR5308, ENS de Lyon, UCB Lyon 1, University of Lyon, University of St-Etienne, Saint-Étienne, France; 4 Laboratory of Infectious Agents and Hygiene, University Hospital of St-Etienne, Saint-Étienne, France

**Keywords:** Disease prevention, Biomedical engineering, Microbiology, Engineering, Materials science, Physics

## Abstract

Can medical face masks be replaced by reusable community face masks with similar performance? The influence of the number of wash cycles, the wash temperature and the use of detergent was evaluated on the performance of one medical face masks (MFM) and ten community face masks (CFM). The performance of the new and washed masks was characterized from the bacterial filtration efficiency (BFE) and the differential pressure (DP). The tests on the new masks showed that the MFM had always better BFE than CFMs. Although two of the CFMs showed a BFE value exceeding 95%, only one can be classified as type I MFM based on both BFE and DP requirements. The influence of the washing parameters was investigated on the MFM and these two CMFs with excellent BFE properties. The parameters had no effect on the BFE of CFMs whilst the MFM exhibited a loss in efficiency when washed with detergent. The DP of masks were not impacted by the washing. The results clearly show that even though a compromise has to be made between the BFE and breathability, it seems possible to manufacture CFMs with performances similar to a type I MFM, without achieving type II requirements.

## Introduction

Respiratory droplets and aerosols can be generated by various expiratory activities like coughing, sneezing and talking. Like SARS-CoV-2, other respiratory viruses are circulated by airborne transmission via droplets and aerosols containing viral particles^1^. During the spread of the SARS-CoV-2, causing the COVID-19 pandemic in 2020, face masks became widely accepted as a means of reducing contamination in indoor environments^2^. The use of masks has been shown to help reduce the spread of the virus since facemasks are primarily used with the intention of preventing the infected wearer transmitting the virus to others (source control)^3,4^. However masks could also protect wearers from contracting COVID-19. Indeed, some studies demonstrated that masks could offer protection to the healthy wearer against infection (protection)^5,6^. Facemasks can be distinguished into respirators, medical and non-medical face masks^7^. Before the Covid-19 pandemic, medical face masks were typically recommended in care services for medical staff. However, in response to the pandemic, governments mandated the wearing of masks in public places which lead to the rapid increase in demand for medical face masks and consequently created tension on their supply. Thus, community face masks or cloth masks were introduced in a bid to address worldwide shortage of medical face masks^8–10^ and are being used in parallel to the medical face masks.

In addition to the shortages, these single use medical face masks are a huge source of waste and contribute to the already existing issue of micro plastic pollution in marine and land environments^11–13^. Consequently, the reusability of masks becomes an increasing subject of interest. Community face masks are therefore seen as a more environmentally friendly option to single use disposable medical face masks as they can be reused several times by washing^14,15^. Although initially designed as single use, studying the feasibility of the reuse of the medical face masks also appears interesting for both environmental and supply reasons. Some studies have shown that medical face masks are capable of being reused after 10 washing cycles^16,17^.

Although the protection of community face masks against small particles is highly variable and typically less than medical face masks^18,19^, their use is still recommended to protect other people from the transmission of the SARS-CoV-2 virus^20^. In this study we seek to determine if some commercially available community face masks have performances that approach that of a standard medical face mask. In addition to this, we also seek to evaluate the influence of three washing parameters on the performance of community face masks, and then to compare their performance to that of a medical face mask, new or washed under the same conditions. The parameters studied include, the number of wash cycles, the water temperature and the possible use of detergent during the washing cycles.

Medical face masks are subject to specific requirements set by standards. In Europe, the performance requirements are described in the standard EN 14683:2019^21^ in terms of minimum Bacterial Filtration Efficiency (BFE), the maximum values for Differential Pressure (DP), the maximum bio burden and requirements for splash resistance. In this work both the medical face mask and community face masks are subjected to the EN 14683:2019, and we focus only on two parameters: the BFE (indicating the filtering capacity of the mask material) and the DP (indicating the breathability of the masks material). In this sense, our study stands out from articles already published on related topics because, although the reusability of medical face masks and community face masks after different washing or decontamination processes has already been published, the number of articles using the bacterial filtration efficiency instead of an efficiency measurement performed with non-biological materials is still very low. In addition, a great originality is thus to use a bioaerosol to measure the filtration efficiency of community face masks after washing according to the standard required for medical masks in order to be able to compare their performance in term of filtration (BFE measurement) and breathability (DP measurement).

## Materials and methods

### Masks tested

Ten community face masks from different French manufacturers were purchased and initially evaluated in this study. The medical face mask that was used in this study is a type IIR medical mask certified according to the European standard EN 14683: AC 2019. Mask references are provided in Supplementary Table S1. Measurements were conducted on five samples of each mask type.

### Bacterial filtration efficiency (BFE)

The evaluation of the BFE was performed according to the EN 14683:2019 standard for the performance of medical masks and using a published procedure^22^. An aerosol stream containing a known charge of *Staphylococcus aureus* ATCC 29213 is generated using an E-flow mesh nebulizer (Pari GmbH, Starnberg, Germany). The counts are expressed in Colony Forming Units (CFU). The culture medium is diluted to obtain a concentration of approximately 5 × 10^5^ CFU mL^−1^ for the tests. The average number of CFUs was maintained on average between 1.7 × 10^3^ CFU and 3.0 × 10^3^ CFU whilst the mean particle size (MPS) is kept at 3.0 ± 0.3 μm. as required by EN 14683:2019. The MPS was calculated as:

$$ MPS = \frac{{\mathop \sum \nolimits_{i = 1}^{6} (P_{i} \times C_{i} ) }}{{\mathop \sum \nolimits_{i = 1}^{6} (C_{i} ) }} $$

where $P_{i}$ is the 50% effective cut-off diameters of each of the six stages of the impactor (ranging from 0.65 to 7 µm), and $C_{i}$ is the number of CFUs grown at the i-th stage when no mask (positive run) is present in the system.

The generated aerosol is then drawn through the aerosol chamber (glass cylinder with a 60 mm diameter and 600 mm length) at a constant flow of 28.3 L min^−1^ by a vacuum pump. The mask samples are clamped between the aerosol chamber and a six stage Andersen cascade impactor. Each of the six stages consists of 400 orifices and a 90 mm plastic Petri dish, containing an agar culture medium, used as impaction plates. Depending on orifices’ diameters, droplets of a given class-size impact on the Petri dish and trigger the formation of a colony of bacteria. The 50% effective cut-off diameters (i.e*.*, the particle diameters corresponding to 50% sampling efficiency) for each of the six stages when operating at 28.3 L min^−1^ are ranging from 7 μm (stage 1), 4.7 μm, 3.3 μm, 2.1 μm, 1.1 μm to 0.65 μm (stage 6).

Each sample measured at least 100 mm × 100 mm and the test area was therefore at least 49 cm^2^ as required by EN 14683:2019. The tests were performed by putting the interior of the mask in contact with the aerosolized bacteria. Each sample was conditioned at 21 ± 5 °C and 85 ± 5% relative humidity for at least 4 h to reach atmospheric equilibrium prior to testing. To evaluate the BFE of a mask, a series of eight successive measurements must be performed. First, a positive-control run is performed without a mask positioned between the cascade impactor and aerosol chamber. Next, five experiments are performed on test samples, changing the mask for each experiment. A second positive control experiment is then performed. Finally, this cycle of eight consecutive experiments ends with a negative-control run in which air is passed, without adding bacteria, through the cascade impactor for 2 min (this serves as a contamination control to verify that the bacteria deposited during the positive run and the test samples came only from the bioaerosol source).

The BFE is calculated as:

$$ BFE = \frac{C - T}{{C \times 100}} $$

where *C* is the mean of the two positive runs of the total CFU of the six plate counts, and *T* is the total CFU of the six plate counts for each test sample.

The petri dishes were incubated at 37 ± 2 °C for 22 ± 2 h. The CFU were counted with an automatic colony counter Scan 4000 (Interscience).

The BFE requirements for different categories of medical face mask according to EN 14683:2019 are indicated in Table 1.

**Table 1:** Performance requirements for medical face masks according to EN 14683:2019.

	Type I	Type II	Type IIR
BFE (%)	≥ 95	≥ 98	≥ 98
DP (Pa cm^−2^)	≤ 40	≤ 40	≤ 60

### Breathability

**Figure 1:**
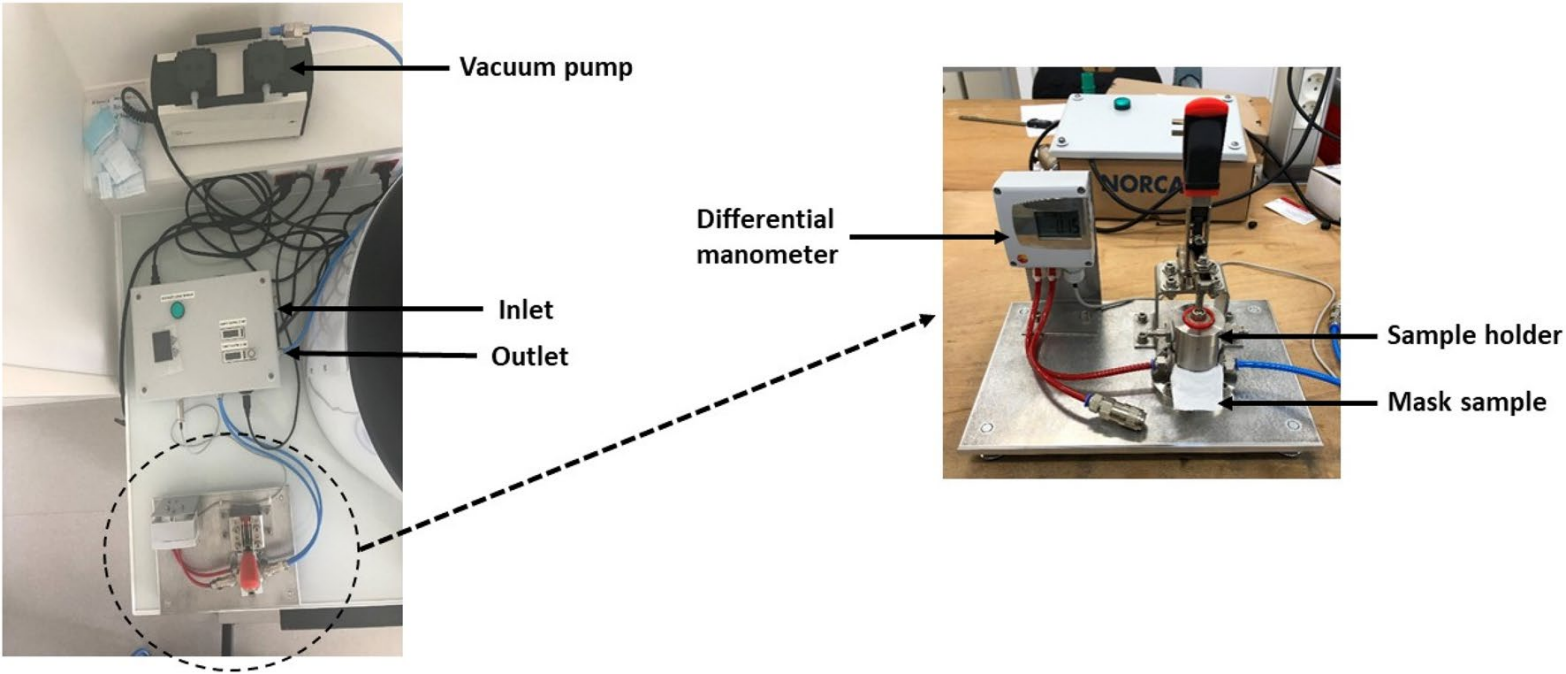
Experimental set-up for the evaluation of the DP compliant with the EN14683:2019 standard test method.

The test for the breathing resistance was performed according to the EN 14683:2019 standard procedure and the experimental set-up is presented in Fig. 1. The mask was attached between two sample holders with a circular cross-section of 4.9 cm^2^, and air passed through the mask at a fixed airflow rate of 8 L min^−1^. The breathing resistance was calculated by measuring the differential pressure drop across the mask material. The differential pressure (DP) was expressed in Pa cm^−2^. The DP requirements for different categories of medical face mask according to EN 14683:2019 are indicated in Table 1. BFE and DP provide valuable information to assess the individual measures of mask effectiveness. However, the dependency of BFE and DP provides a comprehensive filtration quality factor (Q factor in Pa^−1^) and allows for a more robust comparison between filtration media using the following equation:

$$ Q \;factor = \frac{{ - \ln \left( {1 - \frac{BFE}{{100}}} \right)}}{{{\text{DP}} \times { }4.9}} $$

### Microscopic mask characterization

The microscopy analyses were made using a Leica DM LB Microscope with a C Plan lens model. The images were taken with a Bresser MikroCam SP 5.0 at 4× magnification. Scanning Electron Microscopy (SEM) was performed on surfaces of the masks using a JEOL JSM-6500F. Samples were mounted on brass support with double sided carbon tape and coated with 14 nm of gold (Quorom Q 150R ES). Images were taken with beam accelerating voltage of 5 keV.

### Washing procedure

Washing was performed with a domestic washer (Candy Smart CSWS 4852DWE). After a rinse and spin (400 rpm), the masks were dried in open air. Masks were washed 10, 30 and 50 times to evaluate the influence of wash cycles. They were also washed at 30 °C and 60 °C to investigate the water temperature influence and finally, a common commercial laundry detergent (X-Tra Total 3 + 1 Trio-Caps, Henkel Ltd) was used to determine the influence of adding a detergent.

## Results and discussion

### BFE and DP of new masks

**Figure 2:**
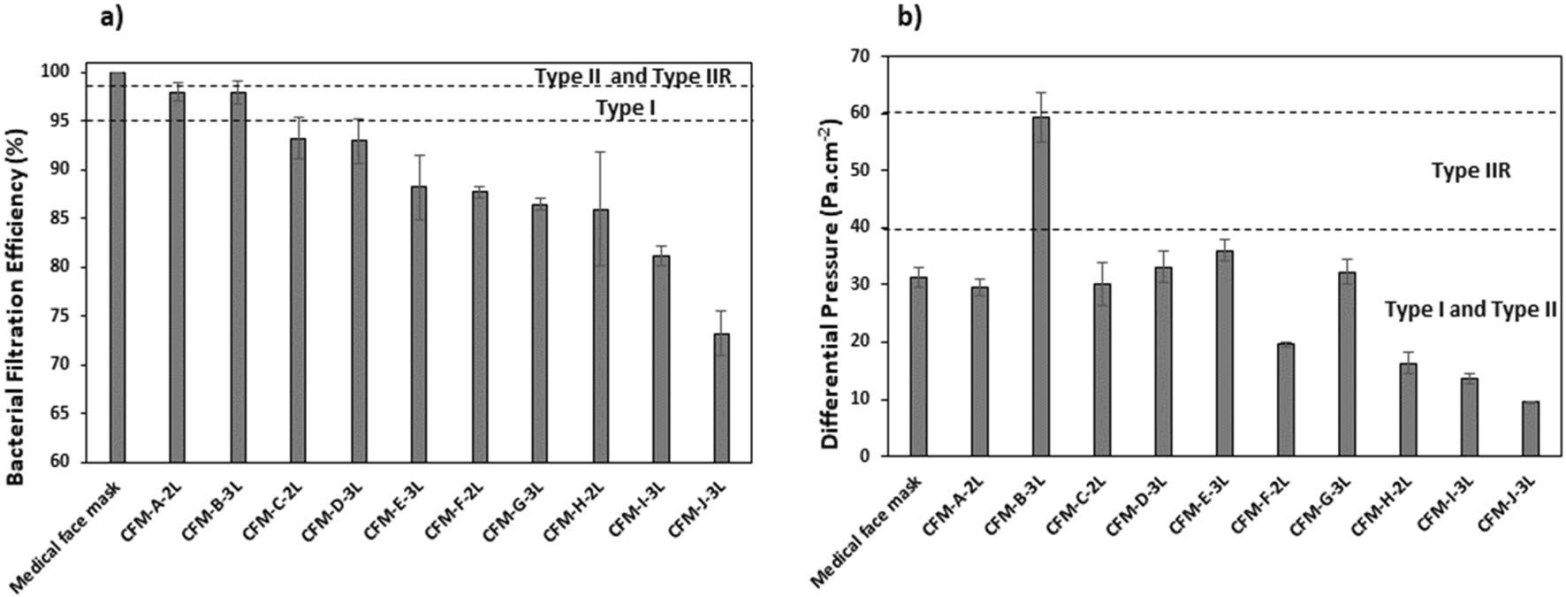
(**a**) Bacterial Filtration Efficiency (%) and (**b**) Differential pressure (Pa cm^−2^) for the medical face masks and community face masks, (average values (N = 5) ± standard-deviation). CFM corresponds to Community Face Masks, 2L corresponds to 2 layers and 3L to 3 layers.

The results of the BFE and the DP of the ten community face masks and the medical face mask when unused are presented in Fig. 2a,b and in supplementary Table S2 (including Q factor values). According to the EN 14683:2019 standard procedure, only the material constituting the masks was evaluated and leakage is not considered in this study. The horizontal dashed lines represent the EN 14683:2019 performance requirement; for type IIR (≥ 98% collection efficiency and ≤ 60 Pa cm^−2^ differential pressure), type II (≥ 98% collection efficiency and ≤ 40 Pa cm^−2^ differential pressure) and type I (≥ 95% collection efficiency and ≤ 40 Pa cm^−2^ differential pressure).

The results showed that all the masks were complaint with the breathability requirement for the various categories of medical face masks (type I, type II and type IIR) except one community face mask (i.e*.* CFM-B-3L) that was not in compliance with a type I or type II mask but in the limit of compliance of a type IIR mask. The medical face mask had the highest filtration efficiency of 99% and was compliant according to type II medical face mask standard. There was a variability in the filtration efficiency of the community face masks with the BFE ranging between 73 and 97%. Only 2 community face masks (i.e*.* CFM-A-2L and CFM-B-3L) had BFE exceeding 95%, the BFE requirement for Type I medical face mask. But all things considered, only one CFM (i.e*.* CFM-A-2L) is compliant with a type I medical face mask requirement, because the breathability of CFM-B-3L is well above the DP limit of 40 Pa cm^−2^. Finally, The MFM show a Q factor at 60.1 kPa^−1^ and the CFMs in the 12.1–28.4 kPa^−1^ range (see Table S2).This result clearly show that a compromise has to be found between the BFE and the breathability to manufacture community face masks with excellent properties. In other words, the key technical challenge for manufacturers is to obtain community face masks with high filtration efficiency but without sacrificing their breathability.

The filtration of aerosol droplets using a face mask is governed by several mechanisms: impaction, interception, diffusion, and electrostatic attraction^9,23^. The contribution of each mechanism to the filtration efficiency of a face mask depends on the materials used (porous structural differences), aerosol droplet sizes, and the operating conditions (temperature, humidity, and air filtration velocity). For aerosol droplets > 1 µm, impaction and interception mechanisms are more important. For small particles < 0.1 µm, diffusion by Brownian motion is the dominant mechanism. When the mask material is charged, electrostatic forces contribute to particle capture especially for particles in the most penetrating particle size (MPPS) range of 0.1–0.5 µm (MPPS zone)^24^ where no mechanism is dominant. For the average particle size of 3 µm required for the BFE, impaction and interception are the most dominant mechanisms.

The performance of the community face masks is influenced by fabric characteristics but the most influential characteristics are currently unclear^25^. Surface characteristics of the material used, such as the pore size disbution (in the 113–981 µm range for CFMs) or the fiber diameter (in the 12–18 µm range for CFMs) are important parameters that potentially influence the performance of the masks^26^. The results of the pore size distribution on CFM and MFM provided in Supplementary Table S3 perfectly show that although an obvious general trend seems to indicate that the higher the pore size, the lower the filtration efficiency, it is difficult to make a robust correlation of the filtration efficiency only from these structural parameters of the masks—, Besides, when it comes to efficiency, it is not just the pores size that are responsible for capturing aerosols, the fiber diameter is also important, especially for masks made of non-woven fabric, as is the case with MFM. Depending on the size and number pores the masks made from braided fabrics, the air flow can increase or decrease when passing through these pores, increasing or not the flow velocity.

**Figure 3:**
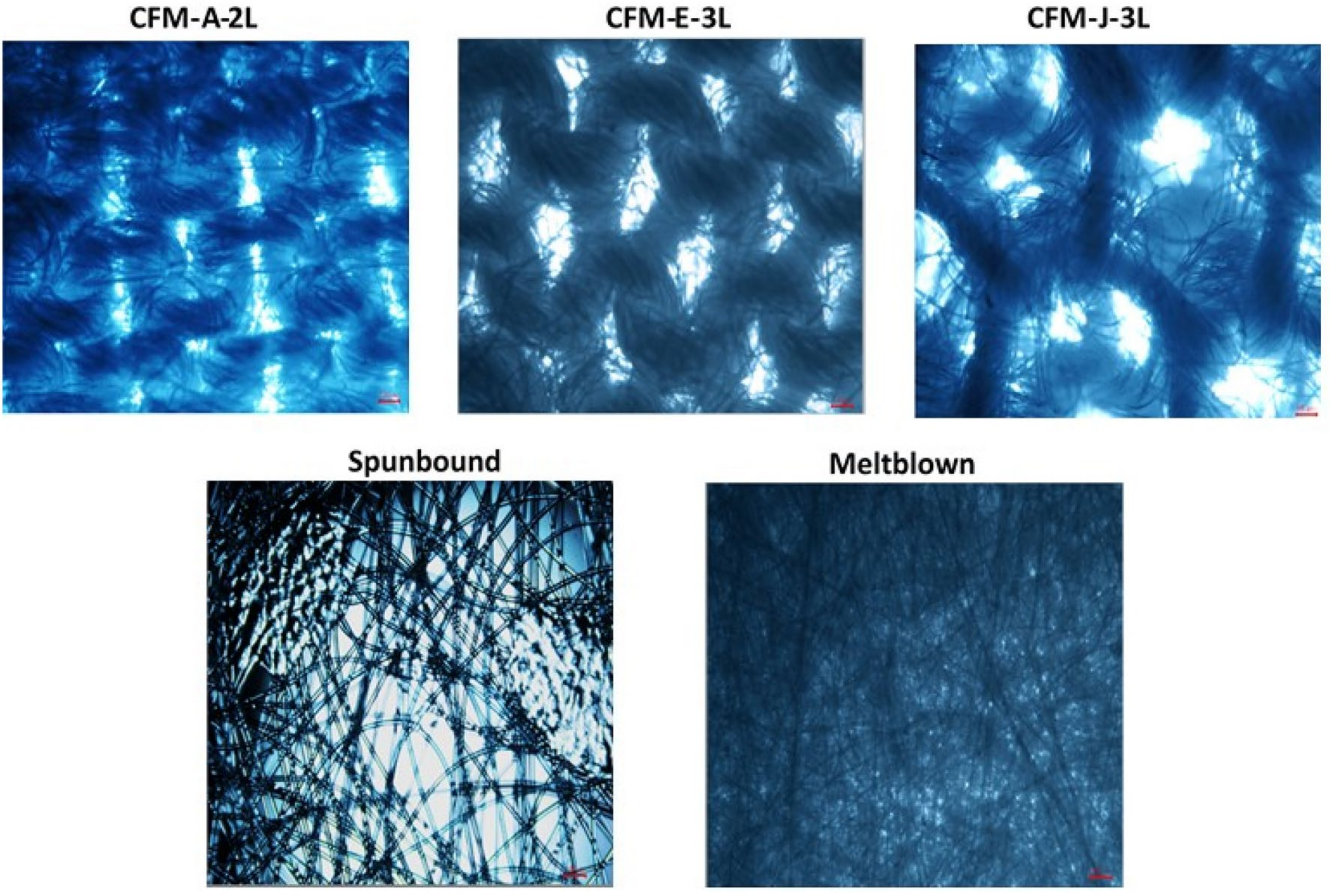
Optical microscopy images of the microscopic structure of 3 community face masks and the medical face mask. (4× magnification and red scale bar corresponds to 100 μm). Optical microscopy images of all CFMs are provided in supplementary Fig.S1.

The representative microscopic images of the community face masks and the medical face mask are shown in Fig. 3. For brevity, only 3 out of the 10 community face masks are represented. Fibrous filter materials are usually comprised of fibers arranged in several ways. For non-woven materials, fibers are randomly oriented whilst woven and knitted materials contain yarns (bundles of fibers) that are interlaced to each other^27^. The pores are formed at yarn interstices for the woven and knitted fabrics whilst they are formed by small spaces between individual fibers in non-woven filters^27^. The spaces in between yarns were considered as the pores for the community face masks. Although the pore shape and size in community face masks were not uniform, we tried to extract quantitative information on the size of the inter-yarn pores by measuring the longest dimension of each inter-yarn pore using ImageJ software. The measurements provided an estimation of the size of an inter-yarn pore in each community face mask: around 150 μm, 330 μm and 900 μm for CFM-A-2L, CFM-E-3L and CFM-J-3L respectively (Fig. 3 and Supplementary Table S3). This could probably explain why CFM-A-2L had the highest filtration efficiency whilst CFM-J-3L had the lowest. Medical face masks are typically made up of 3 layers of non-woven polypropylene fibers (spunbound, meltblown, and spunbound layers). The pore size of the meltblown layer of the medical face mask are estimated to be around 20 μm^28,29^. The small pore size of the meltblown layer compared to the different community face masks could possibly account for its higher filtration efficiency.

In the case of CFMs investigated in this study, the number of layers of the mask wasn’t the most influential parameter. CFM-J-3L which is a 3 layer mask had the lowest BFE whilst CFM-A-2L, a 2 layer mask, had the highest BFE. It seems in this case that layering fabrics with very high pore size doesn’t necessarily improve the BFE or DP.

Based on the results (Fig. 2), 4 categories of masks can be identified:

- Firstly, the medical face mask which has excellent BFE (> 98% (type II)) and low DP (≤ 40 Pa cm^−2^) is compliant with type II medical face mask requirements.
- The CFM-A-2L, which has a good BFE (> 95% (type I)) and low DP (< 40 Pa cm^−2^), that can be categorized as a type I medical face mask.
- The CFM-B-3L, which has a good BFE (> 95% (type I)) but a too high DP (≈60 Pa cm^−2^), that cannot be categorized as a type I medical face mask since this good filtration efficiency was obtained at the expense of poor breathability properties.
- And lastly the 8 other community face masks which had inadequate BFE according to medical face masks requirements (70% < BFE < 95%) with correct DP (< 40 Pa cm^−2^).

To be effective a mask needs to both filter out particles and allow a person to breathe easily. Producing community face masks typically involves a compromise between the BFE and DP and in some cases, having a high BFE comes at the cost of having a high DP leading to low breathability as seen for CFM-B- 3L. According to the results of the community face masks, we demonstrated that it is possible to have community face masks that perform similarly to a medical face mask. Indeed, out of our panel of 10 community face masks, only 1 met the BFE and DP requirements of a type I medical face masks, but could not achieve the type II requirements like the medical face masks chosen in this study. Community face masks are made to be washed and as this may alter the performances, the next part of the study seeks to evaluate the influence of the washing parameters. Only the community face masks that respected the BFE requirement for a type I (CFM-A-2L, CFM-B-3L) were chosen and compared to the medical face mask.

### Influence of wash cycles on the performance of the masks

Firstly, focusing only on the parameters required by EN 14683:2019 standard (i.e*.* BFE and DP), we must underline that the face-fit property of the masks before and after washes has not been tested. It is known that face seal leakage can have a stronger influence on wearers' aerosol and bacteria exposure than filtration efficiency and the shape and face-fit of a mask can change after a vigorous wash and spin of the mask. However, we would like to point out that, by nature, the masks used in this study are not designed to be worn "tight" unlike other types of face masks like FFP2 or KN95 respirators. Therefore, the face-fit property of medical masks is not a property that is required by regulation in the EN 14683:2019 standard (the surgical mask not being designed to be perfectly tight). There is therefore no recognized regulatory protocol for measuring this property on surgical masks (unlike the standard for FFP respirators).

**Figure 4:**
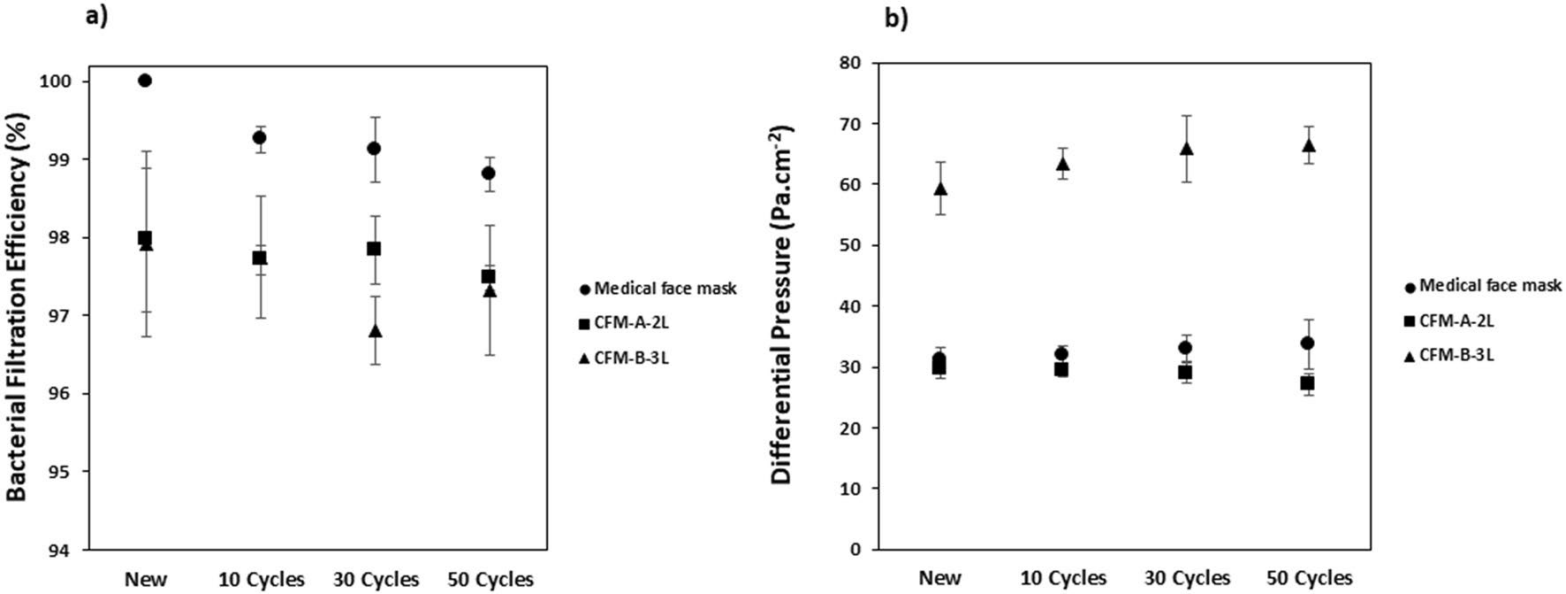
Influence of the wash cycles on the: (**a**) Bacterial Filtration Efficiency (%) and (**b**) Differential Pressure (Pa cm^−2^) on the medical face mask and community face masks. Average values (N = 5) ± standard-deviation.

To evaluate the effect of the wash cycles, the masks were washed 10, 30 and 50 times at 60 °C with the laundry detergent. The results of the BFE and DP are shown in Fig. 4. From the graph (Fig. 4b), it is observed that washing didn’t significantly impact the differential pressure of the medical face mask and the community face masks.

Concerning the community face masks, the washing cycles didn’t impact in a significant manner the BFE and thus they were able to maintain their performance up to 50 washes. This was in accordance to previous study by Sankhyan et al.^30^, who found that community face masks could be washed 52 times without significant loss in particle filtration efficiency. For the medical face mask, the BFE decreased by 1% when the masks were washed but its DP remained constant up to 50 washes. Alcaraz et al*.*^17^ also observed a slight decrease in BFE of medical facemasks when washed but concluded that they could be washed up to 10 times without further degradation of the filtration or breathability properties. The reason for the decrease in efficiency when the medical face mask is washed is as a result of the loss of electrostatic charges which will be explained in “Influence of the use of detergent on the performance of the masks” section.

**Figure 5:**
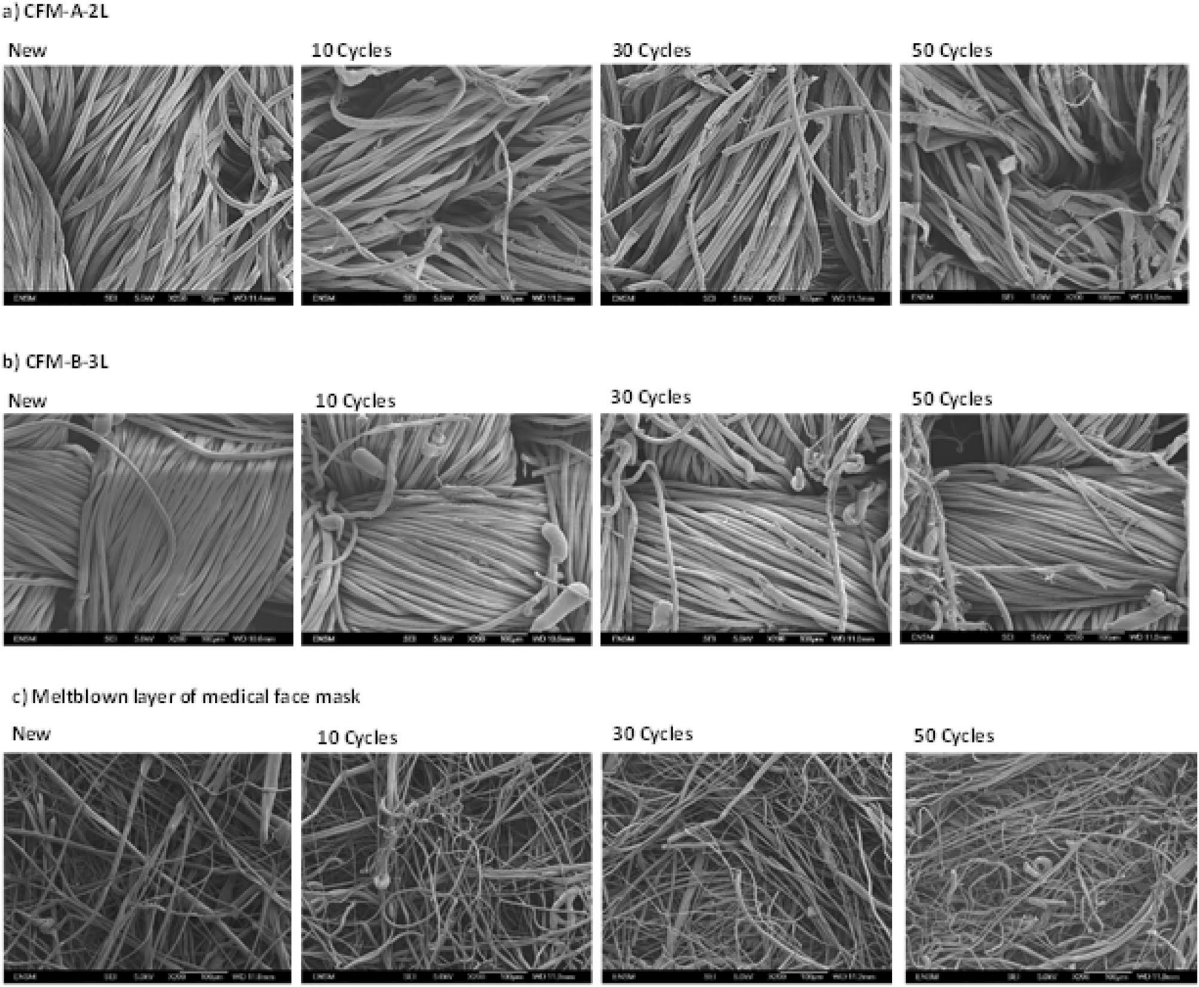
SEM images (200X magnification and scale bar corresponds to 100 μm) of : (**a**) CFM-A-2L, (**b**) CFM-B-3L, (**c**) meltblown layer of the medical face mask; for the new and washed masks subjected to varying number of wash cycles.

SEM images of the new and washed medical face mask (meltblown layer) and the community face masks are presented in Fig. 5. The new community face masks exhibited fiber bundles (yarns) that were globally intact with relatively smooth texture. After 10 washes, there was some liberation of individual fibers from the fiber bundles and there was some deconstruction of the individual fibers which increased slightly after 50 washes (Fig. 5a,b). This however didn’t seem to impact the performance of the masks, as despite the liberation and deconstruction, the fiber bundles remained globally intact. For the medical face mask, very few meltblown fibers exhibited breakages (Fig. 5c).

### Influence of temperature on the performance of the masks

**Figure 6:**
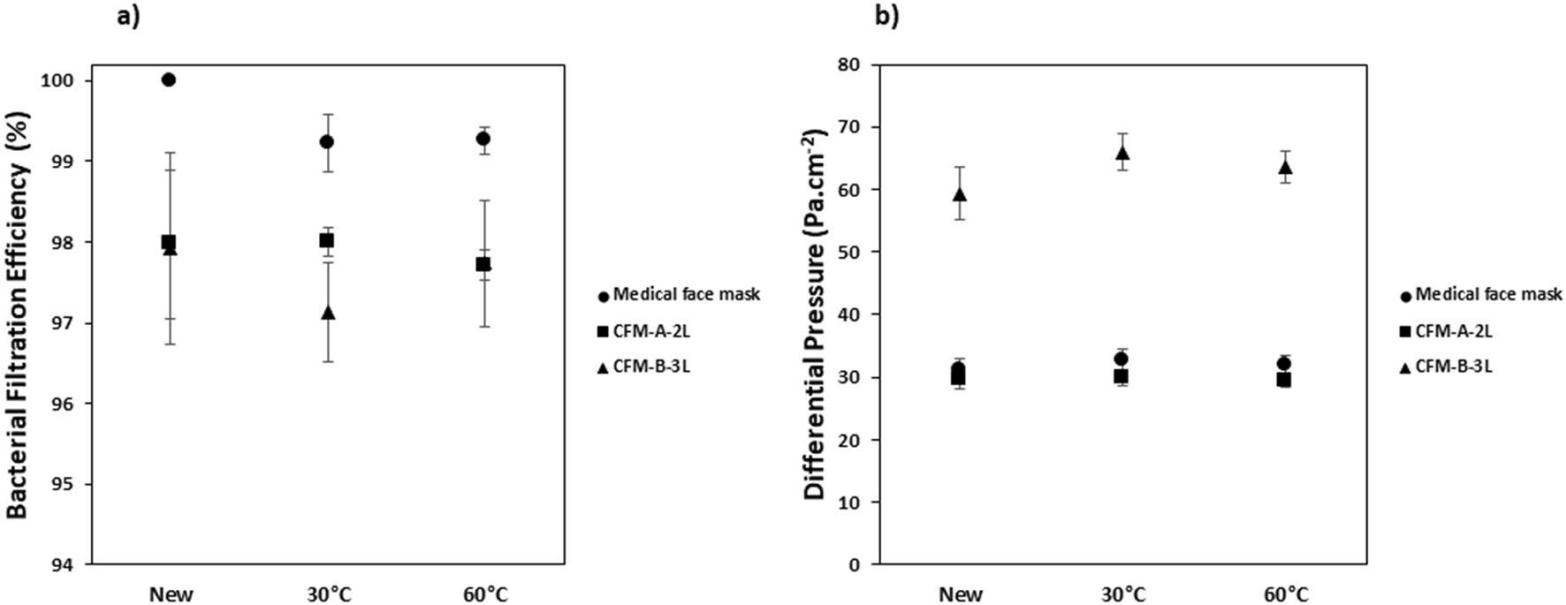
Influence of the wash temperature on (**a**) Bacterial Filtration Efficiency (%) and (**b**) Differential Pressure (Pa cm^−2^) for the medical face mask and the community face masks Average values (N = 5) ± standard-deviation.

The effect of the wash temperature on the performance of the masks was studied by varying the temperature at 30 °C and 60 °C whilst the number of wash cycles was kept at 10 and detergent used for each wash. The results of the BFE and DP for the masks are shown in Fig. 6a,b.

With regards to the community face masks, the temperature didn’t seem to influence greatly their performances (BFE and breathability). The SEM images (see Supplementary Fig. S2a,b) showed similar deconstruction levels of the washed fibers which is attributed to the mask being washed 10 times rather than the temperature. The fiber bundles were globally intact in all cases.

For the medical face mask, there was a decrease in the BFE of the washed masks compared to the new mask, however, the wash temperature didn’t seem to influence its BFE. The meltblown layer of the medical face mask is charged electrostatically by corona effect to increase particle collection efficiency. The charge stability can be affected by temperature. Liu et al*.*^31^ subjected the electret meltblown layer to heat treatment at several temperatures at various times (1–24 h) and noticed that below 70 °C the effect on the filtration efficiency was minimal up until 24 h of treatment but when the temperature was increased to 90 or 110 °C, the filtration efficiency decreased significantly with the increase of the treatment time. They attributed it to the fact that higher temperatures led to higher charge escape/loss which subsequently led to a reduction in electrostatic effect. The temperatures studied in this work were not high enough to impact the charge stability of the electret layer and may explain why there was no impact on the BFE. SEM images (see Supplementary Fig. S2c) also show that the temperature didn’t affect the fiber morphology. Finally, the DP of the medical face mask wasn’t also impacted by the temperature change.

### Influence of the use of detergent on the performance of the masks

**Figure 7:**
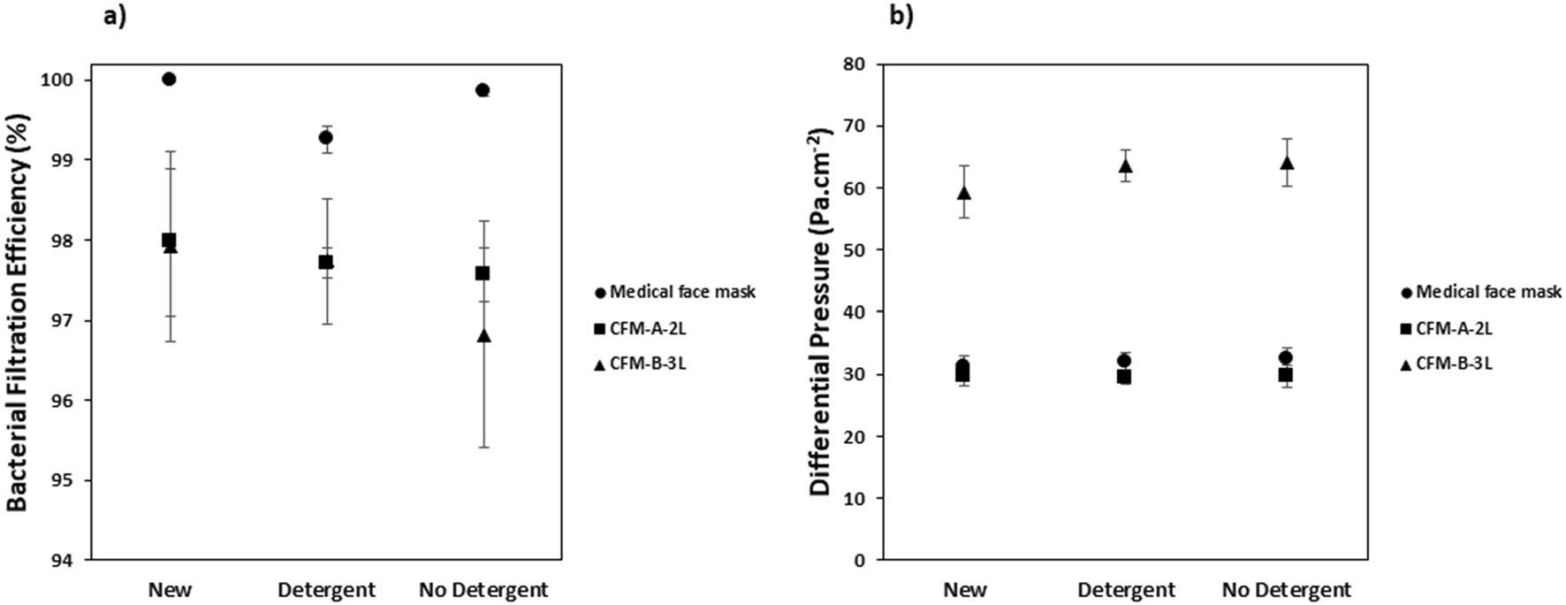
Influence of detergent on (**a**) Bacterial Filtration Efficiency (%) and (**b**) Differential Pressure (Pa cm^−2^) for the medical face mask and the community face masks. Average values (N = 5) ± standard-deviation.

The masks were washed 10 times, at 60 °C with and without detergent to determine the influence of the use of detergent on their performance. The results of the BFE and DP are shown in Fig. 7a,b.

The presence of the detergent didn’t seem to impact significantly the BFE and DP of the community face masks. SEM analysis (see Supplementary Fig. S3a,b) also showed that the fiber morphology was not significantly impacted by the use of a detergent and once again the deconstruction of the fibers was attributed to the number of wash cycles. The fiber morphology of the medical face mask was also not significantly impacted by the use of a detergent as shown in Supplementary Fig. S3c.

**Figure 8:**
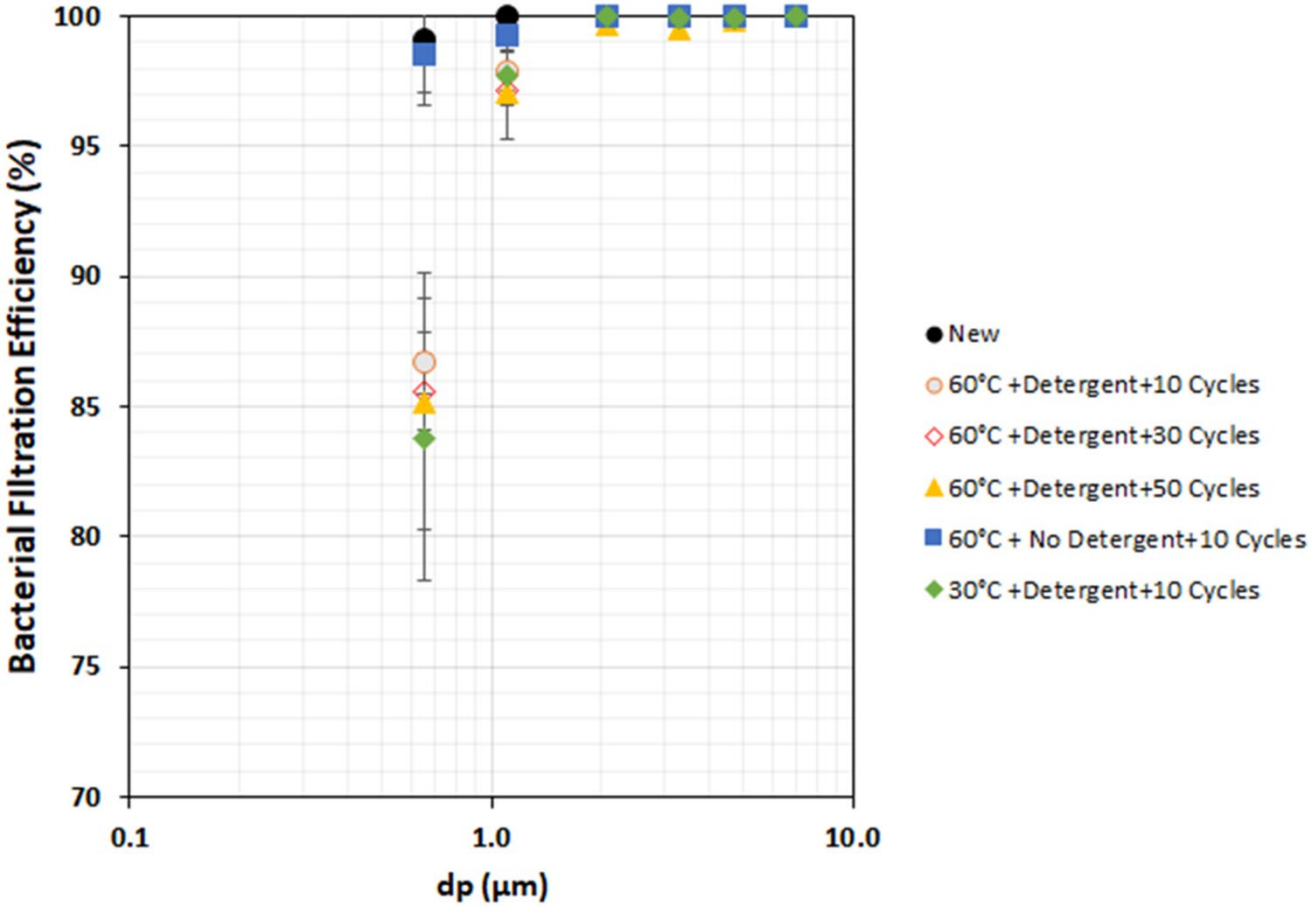
Influence of different washing conditions on the Spectral Bacterial Filtration Efficiency (%) of the medical face mask Average values (N = 5) ± standard-deviation.

Concerning the medical face masks, the BFE for the mask washed without detergent was similar to that of the new mask but the BFE was decreased when the mask was washed with the detergent. This shows that the presence of the detergent is probably responsible for the loss in BFE for the medical face mask. The washing agents present in the detergent are likely to bind to the surface and cause a loss of electrostatic charges of the electret meltblown layer^32–35^. This observation was also highlighted by Charvet et al*.*^16^ and Alcaraz et al*.*^17^. The reduction of efficiency was observed only for the submicron particles (impaction plate collection size between 1.1 and 0.65 μm) as shown in Fig. 8. Inertial impaction and/or direct interception are the dominant particle capture mechanisms for particles > 1 μm but for submicron particle sizes other mechanisms particularly the electrostatic mechanism play an important role.

The loss of electrostatic effects could likely be attributed to the presence of cationic surfactants in fabric softeners. These compounds, notably esterquats, possess excellent antistatic properties and are used to prevent the accumulation of static charges. Thus, the components of detergent have certainly a strong influence on the degradation of filtration efficiency. Therefore the loss of electrostatic charges caused by the detergent tends to reduce filtration efficiency for the submicron particles. Charvet et al*.*^16^ and Alcaraz et al*.*^17^ mimicked the loss of electret effect by discharging a medical face mask in isopropanol. Their results showed that spectral filtration efficiency of a mask discharged by immersion in isopropanol was similar to that of a washed mask.

## Conclusions

The recent COVID-19 pandemic has led to an increase demand for the use of face masks across the globe. Due to the shortages at the early stages of the pandemic and environmental implications of the end of life of single use medical face masks, the reusability of these masks and the use of reusable community face masks is of interest.

With the exception of one community face mask (i.e*.* CFM-B-3L which was in the limit of compliance of a type IIR medical face mask), all the masks tested were complaint with the breathability requirement for the type I and type II categories of medical face masks (i.e*.* DP < 40 Pa cm^−2^). The medical face mask had the highest BFE of 99% and was compliant according to type II medical face mask standard. By contrast, there was a variability in the BFE of the community face masks with the BFE ranging between 73 and 97%. Only 2 community face masks (i.e*.* CFM-A-2L and CFM-B-3L) had BFE exceeding 95% (BFE value corresponding to a type I medical face mask requirement). The variability of performance of the masks in particular the community face masks was attributed to the fabric characteristics specifically the pore size.

The results of the community face masks clearly show that even though a compromise has to be made between the BFE and breathability, it is possible to have community face masks whose performances are similar to those of a type I medical face masks (*e.g.*CFM-B-3L).

To evaluate the influence of the washing parameters, only the masks that respected the BFE requirement for a type I medical face masks (CFM-A-2L, CFM-B-3L) were chosen and compared to the medical face mask. They were washed and dried 10, 30 and 50 times, washed at 60 °C and 30 °C, with and without detergent.

For the medical face mask, though still within conformity of the BFE and DP requirement for a type II mask, washing led to a slight decrease in the BFE (around 1%). It was observed that the presence of the detergent was responsible for this decrease and that it only impacted the collection efficiency of the submicron particles due to the loss of electrostatic charges of the meltblown layer. Washing and reusing medical face masks can be a solution to tackle the supply and environmental implications of medical face masks during pandemic situations. Providing the medical face masks remain in good shape and can be worn comfortably, they can be used in non-medical environments up to 50 times without significant loss of their bacterial filtration efficiency and breathability.

For the community face masks, the various parameters didn’t influence their BFE and DP. Although slight liberation and deconstruction of the fibers was observed during SEM analysis, the fiber yarns were globally intact. Hence, the CFMs can be washed and reused several times without significant loss of performance.

Conclusively, even though the type II medical face mask was the most efficient, based on our panel of 10 community face masks, 10% have performances (as new and after washing) comparable to a standard type I medical face mask.

## Supplementary Information

Supplementary Information.

## Data Availability

The datasets used and/or analysed during the current study available from the corresponding author on reasonable request.
