# Supplementary material for: Impact of washing parameters on bacterial filtration efficiency and breathability of community and medical facemasks
Source: Sci Rep. 2022 Sep 23;12:15853. doi: 10.1038/s41598-022-20354-w (PMC9508161; doi:10.1038/s41598-022-20354-w)
Supplement: Supplementary file 1 — Supplementary Information. [file 41598_2022_20354_MOESM1_ESM.docx]

# SUPPLEMENTARY INFORMATION

| **Trademark** | **Type** | **Reference in article** | **Photo** |
| --- | --- | --- | --- |
| Gyneas (masque en tissue réutilisable) | Community face mask | CFM-A-2L | 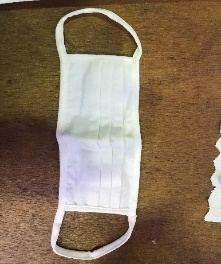 |
| Le Mahieu (Le solidaire) | Community face mask | CFM-B-3L | 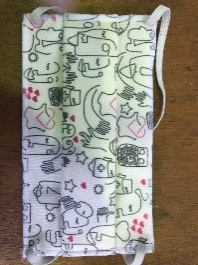 |
| Thuasne (Start Security) | Community face mask | CFM-C-2L | 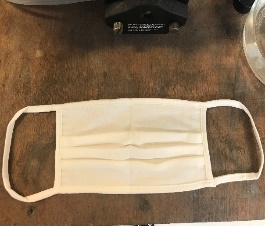 |
| Hero Lab (J17P75) | Community face mask | CFM-D-3L | 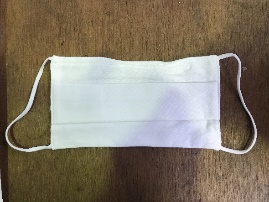 |
| Dim (masque barrière) | Community face mask | CFM-E-3L | 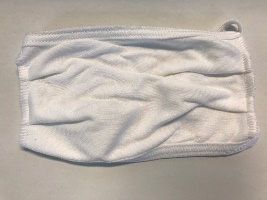 |
| Rozen (Masque 6 ROZEN) | Community face mask | CFM-F-2L | 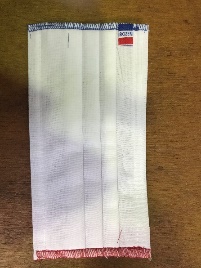 |
| Champion (masque de protection) | Community face mask | CFM-G-3L | 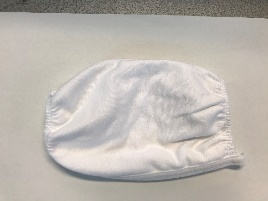 |
| Proneem (VIRAL STOP ®) | Community face mask | CFM-H-2L | 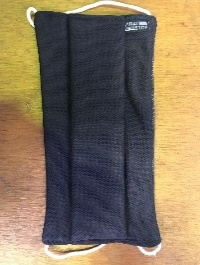 |
| Masque Direct (assortiment fantaisie) | Community face mask | CFM-I-3L | 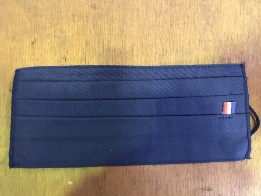 |
| Medimasque (MT-CN) | Community face mask | CFM-J-3L | 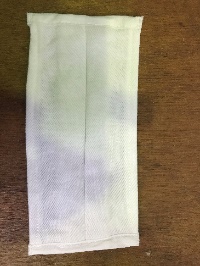 |
| Bioserenity (SKU: 1016-07007-EU) | Medical face mask Type IIR | Medical face mask | 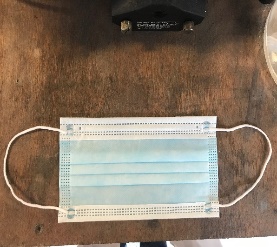 |

**Supplementary Table S1: Medical and community face masks references used in this study. 2L refers to 2 layer masks whilst 3L refers to 3 layer masks**

| Mask Category | Mask |  | BFE (%) | DP (Pa.cm^-2^) | Q factor (kPa^-1^) |
| --- | --- | --- | --- | --- | --- |
| Medical face mask (MFM) | |  | 99.99 ± 0.02 | 31.27 ± 1.80 | 60.1 |
| Community  face mask  (CFM) | CFM-A-2L (mixed fabric) |  | 97.97 ± 0.92 | 26.9 | 26.9 |
|  | CFM-B-3L (mixed fabric) |  | 97.92 ± 1.19 | 13.3 | 13.3 |
|  | CFM -C-2L (mixed fabric) |  | 93.27 ± 2.18 | 18.3 | 18.3 |
|  | CFM -D-3L (mixed fabric) |  | 92.97 ± 2.28 | 16.4 | 16.4 |
|  | CFM -E-3L (100% cotton fabric) |  | 88.22 ± 3.28 | 12.1 | 12.1 |
|  | CFM -F-2L (100% cotton fabric) |  | 87.68 ± 0.66 | 21.8 | 21.8 |
|  | CFM -G-3L (100% cotton fabric) |  | 86.47 ± 0.60 | 12.7 | 12.7 |
|  | CFM-H-2L (mixed fabric) |  | 85.94 ± 5.83 | 24.5 | 24.5 |
|  | CFM-I-2L (mixed fabric) |  | 81.11 ± 1.07 | 24.9 | 24.9 |
|  | CFM-J-3L (mixed fabric) |  | 73.20 ± 2.30 | 28.4 | 28.4 |

**Supplementary Table S2: Performances of the masks expressed by means of BFE (%), DP (Pa.cm^-2^) and Q factor (Pa^-1^). The masks can be classified based on the composition of the fabric (mixed fabrics or 100% cotton fabric) and the number of layers (2L refers to 2 layers ; 3L refers to 3 layers).**

| Mask Category | Mask |  | Fiber diameter  (μm) | Pore size *^b^*  (μm) |
| --- | --- | --- | --- | --- |
| Medical  face mask | Medical face mask Spunbound |  | 25 ± 1 | 100^a^ |
|  | Medical face mask Meltblown |  | 6 ± 3 | 20^a^ |
| Community  Face masks | CFM-A-2L (mixed fabric) |  | 14 ± 4 | 154 ± 13 |
|  | CFM-B-3L (mixed fabric) |  | 12 ± 3 | 218 ± 44 |
|  | CFM -C-2L (mixed fabric) |  | 12 ± 2 | 113 ± 29 |
|  | CFM -D-3L (mixed fabric) |  | 18 ± 3 | 147 ± 27 |
|  | CFM -E-3L (100% cotton fabric) |  | 14 ± 4 | 334 ± 41 |
|  | CFM -F-2L (100% cotton fabric) |  | 12 ± 1 | 621 ± 17 |
|  | CFM -G-3L (100% cotton fabric) |  | 18 ± 4 | 253 ± 47 |
|  | CFM-H-2L (mixed fabric) |  | 14 ± 3 | 548 ± 18 |
|  | CFM-I-2L (mixed fabric) |  | - | - |
|  | CFM-J-3L (mixed fabric) |  | 12 ± 1 | 981 ± 17 |

**Supplementary Table S3: The statistics of fiber diameter and pore size of CFMS and MFM. ^a^ Values obtained from (Ju et al., 2021; Zhao et al., 2020), ^b^ Pore size for community face masks corresponds to gaps between yarns. The spunbound and meltblown are layers of the same medical mask. The masks can be classified based on the composition of the fabric (mixed fabrics or 100% cotton fabric) and the number of layers (2L refers to 2 layers ; 3L refers to 3 layers).**

**
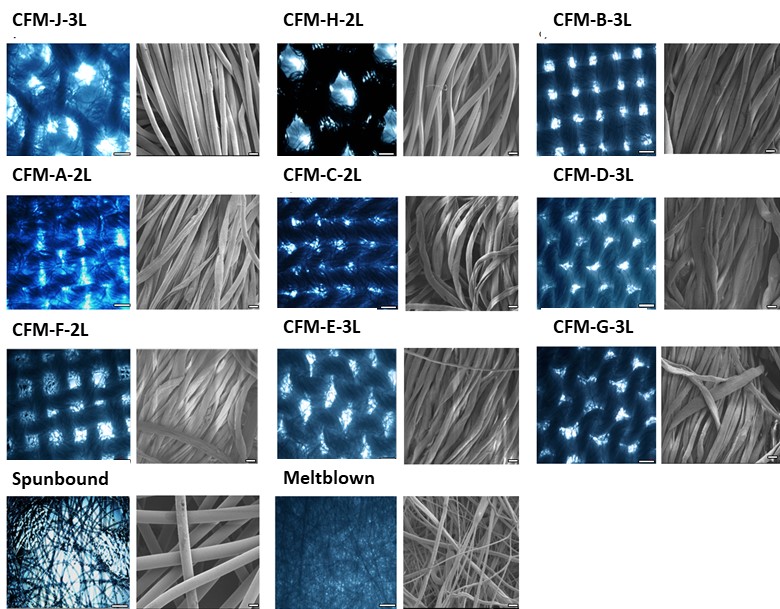
**

**Supplementary Fig. S1**:  **Optical microscopy and SEM images of the microscopic structure of the various masks. Each image is presented in pairs. The left image is the optical micrograph (4X magnification and white scale bar corresponds to 300 μm). The right image is the SEM micrograph (400X magnification and white scale bar corresponds to 30 μm).**

**Supplementary Fig. S2**: **SEM images (200X magnification and scale bar corresponds to 100 μm) of : (a) CFM-A-2L, (b) CFM-B-3L, (c) meltblown layer of the medical mask; for the new and washed masks subjected to two wash temperatures (30°C and 60°C)**

**
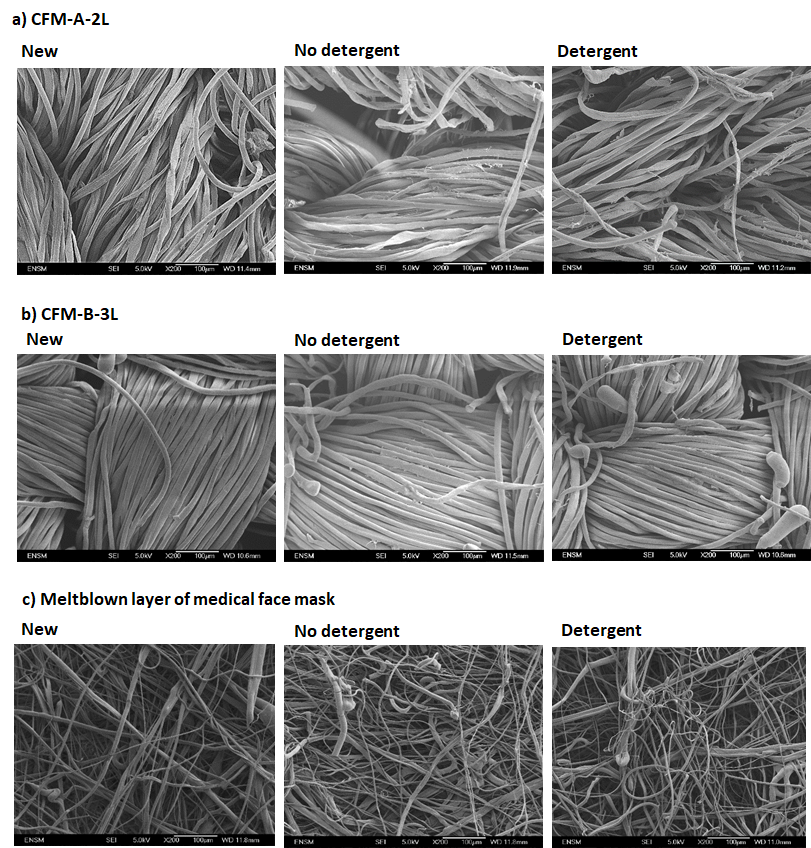
**

**Supplementary Fig. S3**: **SEM images (200X magnification and scale bar corresponds to 100 μm) of : (a) CFM-A-2L, (b) CFM-B-3L, (c) meltblown layer of the medical mask; for the new masks and masks washed with and without detergent**
